# Pathological Plasticity in Fragile X Syndrome

**DOI:** 10.1155/2012/275630

**Published:** 2012-07-02

**Authors:** Brandon S. Martin, Molly M. Huntsman

**Affiliations:** ^1^Center for Neuroscience Research, Children's National Medical Center, Washington, DC 20010, USA; ^2^Interdisciplinary Program in Neuroscience, Georgetown University Medical Center, Washington, DC 20057, USA

## Abstract

Deficits in neuronal plasticity are common hallmarks of many neurodevelopmental disorders. In the case of fragile-X syndrome (FXS), disruption in the function of a single gene, *FMR1*, results in a variety of neurological consequences directly related to problems with the development, maintenance, and capacity of plastic neuronal networks. In this paper, we discuss current research illustrating the mechanisms underlying plasticity deficits in FXS. These processes include synaptic, cell intrinsic, and homeostatic mechanisms both dependent on and independent of abnormal metabotropic glutamate receptor transmission. We place particular emphasis on how identified deficits may play a role in developmental critical periods to produce neuronal networks with permanently decreased capacity to dynamically respond to changes in activity central to learning, memory, and cognition in patients with FXS. Characterizing early developmental deficits in plasticity is fundamental to develop therapies that not only treat symptoms but also minimize the developmental pathology of the disease.

## 1. Introduction

The capacity for appropriate, dynamic, and effective neuronal plasticity is essential for the normal development and function of mature neuronal networks. Neuronal plasticity can be defined as the ability of a neuron or network to functionally alter in response to changes in input or activity. These alterations can occur at the synaptic, neuromodulatory, cell intrinsic, or circuit level and underlie many of the diverse functions within the central nervous system (CNS) such as the development and refinement of connections, learning and memory, regulation of behavior, and cognition. Problems arise in these functions when plasticity mechanisms operate abnormally and neuronal networks improperly develop in response to activity-dependent experience. Accordingly, abnormal neuronal plasticity is a hallmark in many developmental and cognitive disorders including fragile-X syndrome (FXS), the most prevalent inherited cause of intellectual disability and autism spectrum disorder (ASD) [1–3].

FXS is an X-linked, single gene disorder caused by dysfunction in the transcription of the *FMR1* gene that codes for fragile X mental retardation protein (FMRP) [4, 5]. The syndrome results from an irregular expansion of CGG repeats in the 5′ untranslated region of the *FMR1 *gene. Greater than ~200 repeats of this trinucleotide sequence promote hypermethylation and chromatin condensation upstream of the coding region causing transcriptional silencing of *FMR1 *and a subsequent lack of expression of its protein product FMRP [5]. FMRP is expressed in a variety of mammalian tissues but is highly concentrated in the brain and testes [6–10]. In the brain FMRP is located both pre- and postsynaptically and functions mainly as a translational regulator, especially at the synapse [9, 11–15]. It is known to associate with a myriad of neuronal mRNA molecules and an estimated 8% of synaptically targeted mRNA [16–18]. Studies also show that FMRP can function in the nucleus as an mRNA chaperone, binding specific mRNA as part of a ribonucleoprotein (RNP) complex to transport it from the nucleus to the appropriate cytosolic location for protein translation [19]. In humans with FXS, the loss of FMRP results in a variety of neurological symptoms widely associated with imbalances in excitation/inhibition and dysfunctional plasticity in critical brain regions such as the cortex, hippocampus, and amygdala. These symptoms include mild-to-severe intellectual disability, social anxiety and autistic behaviors, increased incidence of epilepsy, attention-deficit hyperactivity disorder (ADHD), and sensory hypersensitivity [2, 20–24]. Shortly after the pathological CGG expansion in the *FMR1 *gene and the absence of its protein product were identified as the source of the disorder [4, 5], an *Fmr1 *KO mouse model of the disease was generated to study the consequences of the loss of FMRP in FXS neuronal networks [25]. These mice display phenotypes consistent with the symptoms of FXS in humans including problems with learning and memory, social interaction, hyperactivity, hypersensitivity, and susceptibility to seizures [19, 25–27]. In addition, early studies of FMRP indicated that the protein was highly expressed in the dendritic shafts and spines of neurons. These observations along with the observation of a higher density and higher proportion of elongated dendritic spines [28–30] in neurons from both humans with FXS and the *Fmr1 *KO mouse led researchers to hypothesize that FXS might primarily be a synaptic plasticity disorder. Subsequently, researchers in Bear's group published the first evidence of pathological plasticity in excitatory hippocampal synapses of the *Fmr1* KO mouse in the form of exaggerated protein translation- and group-I-metabotropic-glutamate-receptor- (GpI mGluR-) dependent long-term depression (LTD). This evidence led to the proposal of the “mGluR theory of FXS” [15, 31] that identifies FMRP as a key downstream regulator of GpI mGluR activation (specifically mGluR5). The theory has been strengthened in recent years by evidence that mGluR5 antagonists or genetic reduction of mGluR5 expression can at least partially rescue both synaptic and behavioral phenotypes in *Fmr1* KO mice [26, 32–40]. However, more detailed examinations into the role of FMRP in controlling activity-dependent protein translation reveal a complex role of the protein in the regulation of activity dependent synaptic, cell intrinsic, and homeostatic plasticity.

The purpose of this paper is to summarize studies that explore the role of FMRP in the regulation of these types of plasticity and their deficits in FXS. We review evidence for the extensive role of GpI mGluRs as well as highlight recently discovered mGluR-independent roles of FMRP. Finally, we discuss how these aberrant processes affect development of neuronal networks in FXS. Our discussion will focus on how pathological plasticity in the disorder effectively reduces the range and stability of responses FXS networks can have in response to changes in activity and/or experience. We emphasize promising areas of study that may advance therapies to alter the course of the pathology and partially restore an effective dynamic range for plasticity in diseased networks. These advances may ultimately reduce the severity of the syndrome and improve responses to current and future therapies for this disease and related autism spectrum disorders.

## 2. The mGluR Theory and Synaptic Plasticity Mechanisms in FXS

Synaptic plasticity is commonly associated with functional changes of pre- and postsynaptic neuronal elements following patterned activity that discretely strengthen (potentiation) or weaken (depression) synapses. FMRP was first connected to synaptic plasticity when researchers identified the protein as upregulated in response to the GpI mGluR agonist 3,5-dihydroxyphenylglycine (DHPG) [41]. This compound induces GpI mGluR-dependent and translation-dependent LTD in the CA1 region of the hippocampus. In this form of LTD, ionotropic glutamate receptors, *α*-amino-3-hydroxy-5-methyl-4-isoxazolepropionic acid (AMPARs), undergo internalization decreasing synaptic strength in response to low-frequency stimulation or DHPG [42]. Soon after the discovery, Huber and colleagues showed the first evidence of pathological plasticity in FXS in the form of enhanced GpI mGluR-dependent LTD in CA1 slices from *Fmr1* KO mice [15]. Because FMRP functions as a negative regulator of translation [12, 14] and is upregulated in response to mGluR activation [41], “the mGluR theory of FXS” was proposed. According to the theory, AMPAR receptor internalization and synaptic destabilizing protein-dependent processes go unchecked in mice lacking functional FMRP. Therefore, protein synthesis related to mGluR activation overall is dysregulated [15, 31, 43]. 

Since the initial proposal of the mGluR theory, mGluR-dependent and -independent synaptic plasticity mechanisms have been thoroughly evaluated in the *Fmr1 *KO mouse. The results of these studies reveal highly region- and modality-specific dysfunction in postsynaptic plasticity mechanisms. In the cerebellum of *Fmr1* KO mice, for instance, mGluR1-dependent LTD is enhanced similar to hippocampal area CA1 [44]. However, N-Methyl-D-aspartic acid (NMDA) receptor-mediated non-mGluR-dependent long-term potentiation (LTP) is not affected in hippocampal circuits in these mice [15, 45–47] revealing the specificity of FMRP for regulating mGluR-dependent plasticity. In other regions such as deep somatosensory cortical layers in which non-mGluR-dependent and mGluR-dependent LTP mechanisms coexist, mGluR-dependent LTP is not enhanced but absent [48]. Furthermore the mGluR5 selective antagonist 2-methyl-6-(phenylethynyl)-pyridine (MPEP) cannot rescue this phenotype in *Fmr1* KO mice [48]. Similar deficits in mGluR-dependent LTP were revealed in the basolateral amygdala of these mice [49]. Although seemingly contradictory to the “overactivation of mGluR mediated protein synthesis” hypothesis put forth by the mGluR theory, these results might be explained by an upregulation of mGluR-dependent processes during development that may have washed out and/or eliminated this type of plasticity from the particular synapse. Network alterations as a result of the loss of FMRP or enhancement of mGluR signaling during development could also explain the attenuation. In the basolateral amygdala deficits in mGluR-dependent LTP were accompanied by decreases in basal synaptic transmission [49]. In accordance with these possible network alterations, similar attenuations in non-mGluR-dependent LTP exist in areas like the anterior cingulate cortex (ACC) and lateral amygdala in *Fmr1* KO mice [50]. The role of FMRP and mGluRs in development is further discussed in the section below. Alternatively, LTP attenuation could result from the upregulation of other proteins normally regulated by FMRP that affect synaptic plasticity. One example is the dendritically located voltage-gated potassium channel Kv4.2, which regulates the induction of NMDA receptor-dependent LTP by theta burst stimulation. This channel is overexpressed in the dendrites of CA1 pyramidal cells in young *Fmr1* KO mice, and these mice show deficits in this type of LTP. Blocking Kv4.2 with heteropodatoxin HpTx2 restores LTP in *Fmr1* KO synapses [51].

Recent studies also characterize deficits in presynaptic plasticity in FXS related to the loss of FMRP in presynaptic terminals. Using isolated sensory-to-motor neuron cocultures derived from *Aplysia*, Till and colleagues (2011) knocked down the *Aplysia* homolog of FMRP (ApFMRP) in either the presynaptic or postsynaptic neuron and evoked LTD with pulses of FRMF amide. They identified enhanced LTD consistent with mGluR-dependent hippocampal LTD if the FMRP knockdown was applied to the postsynaptic cell *or* the presynaptic cell indicating a crucial role of presynaptic protein regulation to regulate LTD [52]. Another study examined presynaptically regulated short-term depression (STD) in *Fmr1* KO hippocampal excitatory synapses. Neurons from *Fmr1* KO mice exhibited enlarged vesicle pools and increased vesicle turnover that correlated with reduced STD when compared to wild-type mice. Consequently, these synapses showed increased responses to replicated high-frequency place field stimuli. These data indicate a strong presynaptic requirement for regulation by FMRP in this type of processing [53].

## 3. Neuromodulatory Endocannabinoid Plasticity in FXS

Region-selective and mechanism-dependent alterations in plasticity in FXS are not exclusive to disruptions in excitatory neurotransmission. Exaggerated signaling through mGluR5 receptors can alter the strength and duration of inhibitory neurotransmission in a form of chemical plasticity of excitatory circuits. GABA release is modulated by both membrane depolarization and through presynaptic receptors that act to reduce the amount of neurotransmitter in the synapse [54–56]. One mechanism involves the synthesis and release (or mobilization) of endocannabinoids—endogenous neuromodulatory lipids that target type 1 cannabinoid receptors (CB1Rs) on the presynaptic terminals of inhibitory interneurons [55]. Activation of Gp1 mGluRs enables the mobilization of endocannabinoids in the postsynaptic neuron and retrogradely modulates GABA release through a mechanism known as depolarization-induced suppression of inhibition (DSI) [57]. The binding to CB1Rs on the presynaptic terminal of the inhibitory interneuron leads to a transient suppression of voltage-gated calcium channel activity thus inhibiting GABA release. These mechanisms require heightened neuronal activity—an environment that exists in brain circuitry of *Fmr1* KO mice [7, 58]. In the CA1 region of the hippocampus, enhanced mGluR signaling leads to excessive endocannabinoid mobilization in *Fmr1 KO *mice and enhanced suppression of inhibitory transmission [59]. This increase in the suppression of inhibition is proposed as a potential contributor to the hyperexcitable phenotype in the *Fmr1 *KO hippocampus [59]. In hippocampal circuitry, endocannabinoid modulation of DSI likely involves specific inhibitory circuits relegated to perisomatic targeting interneurons [60]. Therefore, with respect to endocannabinoid mobilization in the FXS brain, the loss of FMRP may selectively affect specific inhibitory circuits and leave other circuits intact.

In the cerebral cortex endocannabinoid mobilization can retrogradely modulate the release of presynaptic GABA [61] or act to hyperpolarize a specialized type of inhibitory interneuron known as the low threshold spiking (LTS) cell through endogenous autocrine release [54]. In this mechanism, sustained action potential activity activates voltage-gated calcium channels for the influx of calcium in LTS interneurons that triggers the synthesis of endocannabinoids. The binding of endocannabinoids to CB1Rs expressed within the same neuron function to activate G-protein-coupled inward-rectifying potassium (GIRK) currents, resulting in a prominent hyperpolarization that can last for several minutes [54]. This mechanism is known as slow self-inhibition (SSI) and is specific to cortical LTS interneurons [54, 62]. While there is no known abnormality in FXS for this type of interneuron, it is likely affected in FXS. Both Group I and Group II mGluRs selectively activate cortical LTS interneurons causing sustained action potential firing during drug application [63, 64]. Our studies show that DHPG-induced mGluR activation of LTS interneurons is abnormal in *Fmr1* KO mice [65]. mGluR activation of LTS interneurons in the developing and mature brain is critical for the proper synchronization of cortical excitatory neurons at behaviorally relevant frequencies [63, 64, 66, 67]. Therefore, alteration of mGluR signaling in this specific type of interneuron likely has wide-reaching ramifications in developing and mature cortical networks.

## 4. Intrinsic and Homeostatic Plasticity in FXS

The extent of pathological plasticity related to excessive GpI mGluR signaling is not restricted to the synapse. Upregulation of mGluR-regulated processes can fundamentally alter the excitability of the neuron and subsequently modify network dynamics. In 1998, Wong and colleagues demonstrated that GpI mGluR stimulation with the agonist DHPG increased epileptiform burst frequency and duration in hippocampal area CA3 pyramidal cells [68, 69]. These bursts are related to ictal discharges during seizures [70]. They showed that the increase in burst duration was protein synthesis-dependent because the prolongation of bursts persisted after agonist washout and did not occur in the presence of the protein synthesis inhibitors anisomycin or cycloheximide [69]. Subsequent investigations have further characterized the GpI mGluR-mediated prolonged discharges to occur in area CA3 without blockade of GABA receptors [71, 72], to resist generation by repeated glutamatergic synaptic activation alone (without exogenous agonist, i.e., DHPG) [73], and to require GpI mGluR-dependent mRNA translation by way of the tyrosine kinase-extracellular signal-regulated kinase (ERK) 1/2 signaling pathway [72, 74]. Studies implicate a key voltage-dependent cation current, I_mGluR(V)_, as a mechanism underlying GpI mGluR-dependent epileptogenesis [74, 75]. I_mGluR(V)_ upregulation requires phospholipase C *β*1, outlasts acute mGluR activation, is protein synthesis-dependent, specifically tyrosine kinase-ERK signaling pathway-dependent, and blockade of the current suppresses DHPG-induced epileptogenesis [74]. I_mGluR(V)_ is a persistent current with a threshold of around −65 mV (near resting potential) and reversal potential at approximately −10 mV [74, 76, 77]. Its activation induces long bursts of action potentials (up to 12 seconds) and creates a bistable resting membrane potential in CA3 pyramidal cells. Together these properties along with the recurrent synapses of the CA3 network produce epileptiform discharges when I_mGluR(V)_ is sufficiently activated [74, 75].

Since FMRP is a central regulator of the ERK pathway [72, 74, 78], its presence is crucial to the control of I_mGluR(V)_ activation. Although synaptic activation of GpI mGluRs alone is insufficient to produce I_mGluR(V)_-dependent synchronized bursting in wild-type CA3 pyramidal cells [74, 79], these discharges can be induced in *Fmr1* KO hippocampal slices by upregulating glutamatergic transmission alone (via GABAa receptor blockade) without the addition of a GpI mGluR agonist like DHPG [74, 80, 81]. In effect, the *Fmr1* KO CA3 pyramidal cell is predisposed to a persistent activation of I_mGluR(V)_ that thereby renders the CA3 network susceptible to plastic adjustments in favor of the generation of prolonged epileptiform discharges [74, 80, 81]. Moreover, this maladaptive plasticity may be accentuated further by the GABAergic deficits known to exist in the mature *Fmr1 *KO hippocampus and elsewhere [7, 82, 83]. Taken together, this combination of deficits produces a network that is likely more susceptible to hyperexcitability and epileptogenesis when faced with relatively normal increases in neuronal activity. Evidence from audiogenic seizure behavioral assays suggests that this susceptibility underlies decreased seizure threshold in *Fmr1* KO mice and seizures in humans with FXS [27, 36].

The lack of FMRP in the above case likely perturbs the homeostatic balance that translational repression would have on the local increase in expression of I_mGluR(V)_ allowing excitation to spread unchecked from the synapse to the whole cell and network. FMRP has recently been implicated in another form of homeostatic plasticity, *mGluR-independent*, retinoic-acid- (RA-) dependent synaptic scaling. Synaptic scaling is an increase in synaptic strength in response to a prolonged reduction in activity. Observation of the phenomenon is usually achieved *in vitro* by blocking synaptic activity and NMDA receptors with tetrodotoxin (TTX) and aminophosphonovalerate (APV), respectively. This type of plasticity is fundamental for perfecting neuronal connectivity, stabilizing the network, and setting the operational range for coding by the network [84, 85].

RA synthesis increases in response to reductions in activity and crucially regulates synaptic scaling by inactivating the translational repressor retinoic acid receptor *α* (RAR*α*) [86]. The release of this repressor allows synthesis of key proteins required to strengthen the synapse [87]. These proteins include the AMPAR components GluR1 and GluR2 which serve to strengthen the synapse in response to activity-dependent RA signaling [87, 88]. Using hippocampal cultures Soden and Chen (2010) determined that FMRP is required for the RA-dependent increases in AMPAR insertion and synaptic strength. Cultures from *Fmr1* KO mice did not show synaptic scaling but viral introduction of FMRP into *Fmr1* KO cells restored synaptic scaling in response to RA. The researchers subsequently used modified FMRP constructs to show that FMRP binding to mRNA is required to reduce elevated protein synthesis and induce scaling by upregulation of AMPARs. In addition they showed that homeostatic scaling requires FMRP-directed interaction with actively translating ribosomes to upregulate AMPAR insertion. Therefore both FMRP binding to mRNA and FMRP-directed interaction with active ribosomes are necessary to upregulate AMPARs in synaptic scaling, while mRNA binding alone is sufficient to downregulate increased protein synthesis (presumingly resulting from GpI mGluR overactivation) [88].

These data therefore support a dual role for FMRP, first, in translational suppression at the synapse regulating mGluR-dependent Hebbian plasticity and, second, in homeostatic translation induction in response to decreases in activity. Although these roles seem contradictory, they are performed by different mechanisms and therefore may act in concert to dynamically regulate networks. Since homeostatic plasticity sets the dynamic coding range of the network and stabilizes and balances activity levels [84, 85], deficits in this plasticity could further weaken already compromised Hebbian plasticity at the synapse in FXS by failing to maintain the strength of established connections [88]. Deficits in plasticity are regionally diverse in FXS (see above); therefore interactions between synaptic and homeostatic mechanisms in different regions likely result in varied alterations in the operational range and coding capacity of the network. In the hippocampus, for instance, enhanced mGluR-dependent LTD lowers coding capacity, and faulty homeostatic mechanisms may exacerbate the problem by shrinking the range of activity to which the network would respond. These issues loom larger when we consider an understudied question in the field: how does aberrant plasticity effect the establishment of compromised FXS networks during development? The answers to this question may provide the necessary insight to develop therapies to lessen the severity of the disease through earlier therapeutic intervention and improve lifelong response to treatments.

## 5. Pathological Plasticity in the Development of FXS Networks

The same kinds of plasticity that govern learning, memory, and cognition in the mature network refine the developing network especially during developmental critical periods. Critical periods are discrete time windows during which the connectivity of a developing network can be adjusted and refined [84, 89, 90]. After the critical period closes, opportunities for extensive network alterations drop tremendously, and experience no longer modifies networks to the same extent [84, 89, 90]. We can empirically recognize these decreases in plasticity capacity with age when we try to learn new skills like playing a musical instrument after childhood and young adulthood. Critical periods have been especially studied in the cortex and present in a hierarchical fashion [90]. That is, primary cortical sensory areas tend to have earlier critical periods than integrative cortical centers. Disruptions during the critical period of network development can drastically and *permanently* alter the ability of the network to accurately respond to normal activity resulting in irregular sensory processing. Hubel and Wiesel's pioneering studies in the primary visual cortex of the cat notably indicated the permanent loss of visual acuity in adulthood of an eye deprived of experience in the visual critical period [91]. In humans, congenitally deaf children receiving cochlear implants develop hearing and speech most successfully if they receive the implant before ~7-8 years old [92]. Similarly, monaural deprivation in rats only results in interaural imbalance and tonotopic cortical map reorganization if deprivation occurs in young animals versus adult rats [93]. Critical periods for cortical map development have been characterized in the somatosensory system of rodents [94, 95] and the human [96] and animal visual system as well (reviewed in Berardi et al. [97]).

Patients with ASD of various etiologies generally experience deficits in sensory processing consistent with disrupted critical periods leading some to postulate that autism is a “critical period disorder” [89]. FXS is no exception. For example, tactile defensiveness, or hypersensitivity to a normally mild stimulus, is common in FXS [98, 99], and ocular dominance plasticity in response to monocular deprivation is disrupted in *Fmr1* KO mice [26]. Although not yet extensively studied, several lines of evidence indicate that the pathological plasticity mechanisms and associated deficits discussed in this paper are prime candidates to affect critical periods of FXS network development.

Firstly, FMRP and GpI mGluRs are expressed early in development and participate in activity-dependent processes. FMRP is expressed embryonically in the human and mouse [6, 10, 25, 100–102], and its expression in sensory cortex is regulated by neuronal activity, that is, whisker movements [103, 104]. As detailed above, FMRP has dual pre- and postsynaptic roles at the synapse to regulate activity-dependent plasticity. GpI mGluRs are also developmentally expressed in rodents and humans [105, 106]. Besides activity-dependent synaptic plasticity, these mGluRs regulate many early developmental processes including cell proliferation and survival of neural progenitors [105, 107, 108] and laminar organization of developing cortex through expression in cortical plate Cajal-Retzius cells [109].

Secondly, synaptic balance in FXS networks is faulty. The range and dynamics of plastic mechanisms are severely compromised in the ability to code experience/activity-dependent changes (Hebbian) and maintain those changes (homeostatic) as described above. In addition, either subsequently to or concurrently with excitatory transmission problems, GABA network maturation is crucially disrupted in a region-specific manner (reviewed in Paluszkiewicz et al. [110]). Key regions such as the hippocampus, cortex, striatum, and amygdala display up- or downregulations of GABA receptors, glutamic acid decarboxylase (GAD65/67), GABA transporters, and GABA synthesis and release depending on the particular network [7, 83, 111–114]. Importantly studies show that critical period plasticity is defined by excitation/inhibition balance in developing networks [115, 116]. Specifically, GABAergic transmission is important with regard to initiation, prolongation, and termination of the critical period [116, 117]. Defective temporal and spatial interactions between maturing excitatory and inhibitory networks then could easily modify the time course of critical periods.

Thirdly, recent evidence reveals critical period problems in *Fmr1* KO mice that could be related to dysfunctional plasticity resulting from the loss of FMRP and/or dysregulated mGluR mechanisms. For instance, *Fmr1 *KO mice have abnormal ocular dominance (OD) plasticity. When challenged with brief monocular deprivation (3 days) starting at postnatal day 28 (P28), wild-type mice display depression in the visual responses from the deprived eye followed 4 days later by potentiation of responses from the nondeprived eye. *Fmr1* KO mice instead show immediate potentiation of nondeprived eye responses and insignificant deprived eye depression [26, 118].

In rodent layer IV somatosensory (barrel) cortex, the critical period for thalamocortical plasticity normally occurs in the first postnatal week (through P7), with NMDA-dependent LTP from thalamocortical afferents peaking at around postnatal day 4 (P4). In *Fmr1* KO mice, this critical period is delayed past P7 with LTP levels remaining elevated into the second postnatal week before quickly dropping to wild-type levels by adulthood [103]. The barrel cortex normally develops a stereotypical somatotopic map of thalamic inputs that receive afferents from and respond to individual rodent vibrissae during the first postnatal week [119]. This process appears to proceed as planned in layer IV for map arrangement except for a delay in the reorganization of cells. Normally cell bodies concentrate in the barrel septa (the border between barrels) versus the barrel hollow at P7 but this process is deficient at this time period in *Fmr1* KOs, and septa cell density is low [120]. Perhaps significantly, the normal close of the critical period (P7) coincides with an elevation in FMRP expression in wild-type mice that is obviously absent in the *Fmr1 *KO [120]. Synaptic proteins downstream of GpI mGluRs and NMDARs such as PLC-*β*1 and SynGAP, respectively, are downregulated at this time point in *Fmr1 *KO cortex as well [120]. Both of these proteins' mRNAs are targets of FMRP [18].

Subsequently in layer IV, dendritic localization at the end of the second postnatal week in *Fmr1 *KO mice is delayed with more dendrites remaining in the barrel septa at around P14 instead of concentrating in the barrel hollow. Furthermore morphology of those dendrites skews preferentially toward immature filopodia versus the mature mushroom head phenotype as has been reported in mature cortex and hippocampus in *Fmr1* KOs [120–122]. Also in layer IV at P14, there exists a decreased excitatory drive of local fast-spiking (FS), parvalbumin-positive interneurons that persists into adulthood [58].

Following these layer IV perturbations, a succeeding critical period in the refinement of layer IV to layer II/III connections is affected in *Fmr1* KO mice [123]. Layer IV to layer III ascending connections are weakened at P14 with scattered axonal arbors similar to diffuse dendritic arbors in layer IV at this time point [120]. In addition normal layer IV to layer III synaptic depression in response to activity deprivation does not exist [123].

Critical period plasticity in the cortex is temporally progressive not only from primary areas to integrative areas but also from input layers (layer IV) to integrative (layer II/III) and output layers (layer V/VI) within the same cortical area. Based on this concept and the limited evidence of critical period alterations in the somatosensory cortex in *Fmr1* KO mice, we might predict that as network development progresses, abnormalities caused by plasticity deficits can either compound, normalize by way of compensatory mechanisms, or suspend network development altogether. In fact we probably observe a mixture of these phenomena in FXS. Studies indicate that many of the developmental phenotypes just described normalize by adulthood and thus may simply represent developmental delays. For example delayed increases in LTP in layer IV barrel cortex return to wild-type levels by adulthood (P21) [103]. Layer IV to layer III connections eventually normalize in the mutant mouse [123]. Even some behavioral phenotypes diminish or disappear with maturity including increased seizure susceptibility in *Fmr1* KO mice [124] and epilepsy in humans with FXS [3, 20]. However some phenotypes observed during critical period maturation remain in adulthood, notably deficient excitatory drive of inhibition [58], dendritic morphological immaturity [120–122], and learning and memory deficits [25, 26].

**Figure 1:**
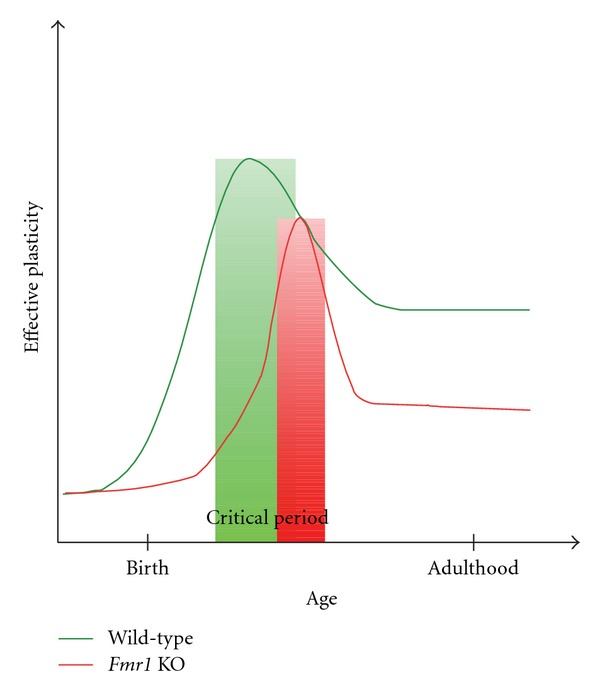
General model of effective plasticity in wild-type versus *Fmr1* KO mice. In primary somatosensory cortex of wild-type mice the capacity for effective plasticity increases rapidly from birth, peaking during the critical period of network development and then normalizing into adulthood. *Fmr1* KO mice display a delay in the increased expression of plasticity mechanisms [103] that normalizes at approximately the same developmental time point as wild-type mice [103, 123]. However, persistent deficiencies in plasticity such as dentritic spine dynamics [122] compromise effective plasticity throughout network development in *Fmr1* KO mice.

Based on the dynamics of plasticity in *Fmr1* KO mice discussed above, we can propose a general temporal model of effective plasticity in which critical periods (at least those of primary cortical areas) are delayed and restricted in FXS (Figure 1). In the FXS brain, even within critical periods though, plasticity is inefficient, compromised by persistent deficits. Then, as the network approaches maturity persistent deficits acting on compromised networks result in decreased capacity for effective plasticity in FXS. Mechanisms responsible for deficits in developmental and postdevelopmental time periods likely have similarities and differences. Then in order to fully understand how the lack of FMRP affects network development, we must discern what plasticity mechanisms are employed during development, the nature of those mechanisms, and the time point at which those mechanisms are crucial for proper network maturity. 

We know that both FMRP and Gp I mGluRs are developmentally expressed and regulated [103, 105, 106, 123], yet little to no studies focus on the role of mGluR-dependent processes during early developmental time points in FXS. Ample evidence shows that pharmacological reduction of GpI mGluR function or genetic reduction of mGluR expression can rescue FXS phenotypes [26, 40, 125] (see http://www.clinicaltrials.gov/). In the case of OD plasticity, *Fmr1* KO mice heterozygous for a knockout of mGluR5 (*Grm5 +/−*), and therefore expressing a 50% reduction in mGluR5 protein, showed the same response as wild-type mice to monocular deprivation [26]. Similar rescues of deficits in spine morphology, increased basal protein synthesis, fear extinction, audiogenic seizures, and learning and memory deficits have been successful [2, 32–34, 36, 37, 39]. However, these experiments, including OD plasticity, focus almost exclusively on alterations in adult animals. Genetic reduction of mGluR5 shows similar effects as pharmacological reduction, but the contribution of developmental versus acute alterations in function cannot be determined by analysis at a single time point.

The role of mGluRs is likely important and unique at early developmental periods in FXS. In hippocampal area CA1 at least, GpI mGluR-mediated LTD undergoes a developmental switch from presynaptically mediated, protein synthesis-*independent* plasticity to postsynaptically mediated protein synthesis-*dependent* plasticity involving internalization of AMPARs. This switch occurs between P8 and P21 which corresponds to the time period of major critical periods in cortical development including primary somatosensory, auditory, and visual [97]. Although GpI mGluRs and FMRP are expressed in these regions during these early periods, no studies to date have elucidated the mechanism of interaction or investigated possible changes in FXS. Given the success of GpI mGluR inhibition to rescue phenotypes and improve symptoms in FXS [26, 40, 125], one might expect that inhibition of this transmitter system may show similar results at early developmental time points. However, Cruz-Martín and colleagues (2010) demonstrate that this is not necessarily the case. In cortex of *Fmr1* KO mice at 2 weeks of age, they observed a delay in spine maturation and increase in dendritic spine turnover. Application of an mGluR5 antagonist did not rescue this phenotype but instead *increased* spine length and motility, an effect directly opposite of that observed in older animals [26, 121]. Whether or not the differences are related to a mechanistic switch of mGluR function downstream of the receptor is unknown, but the evidence indicates that seemingly similar dysfunctional phenotypes (i.e., dendritic morphology) can have different or additional etiologies depending on the developmental time point investigated. Furthermore, reduction of mGluR activity early in development may be deleterious rather than helpful in patients with FXS.

Elucidating the early role of FMRP in developmental plasticity mechanisms in FXS is therefore essential to understanding how the loss of the protein modifies networks and how to improve those negative modifications through targeted treatments. These investigations should not be limited to mGluR mechanisms. Other mechanisms discussed in this paper may also play a role in early development. Endocannabinoid-mediated enhancement of inhibition is developmentally regulated [126] and could play a role in decreased or delayed cortical connectivity in *Fmr1 *KO. Furthermore mGluR regulation of LTS interneurons may be disrupted as described above which could affect the fine tuning of cortical circuits in development [65, 67]. These abnormalities result in faulty synchronization of synaptic inhibition and in turn disrupt DHPG-induced action potential synchronization in cortical pyramidal neurons [65]. FMRP-regulated homeostatic mechanisms may participate early in development as well to hone network connectivity [87, 127]. Although not discussed in detail in this paper (see Paluszkiewicz et al. 2011 [110]), GABAergic regulation of development likely plays a crucial role in regulating early developmental plasticity. GABAergic inhibition and the balance of excitation and inhibition define critical periods [90, 116, 117]. In particular, the developmental maturation of parvalbumin (PV) positive FS interneurons and their connectivity may regulate critical periods, at least in visual cortex [128, 129]. Since this connectivity develops defectively in *Fmr1* KO mice as indicated by a decreased excitatory drive of these PV positive cells at a relatively early developmental time point (P14) [58], determining the integration of inhibitory and excitatory plasticity represents an important target for future research. Relatively new genetic tools in the form of *Fmr1* conditional KO and conditional “ON” mice that utilize the Cre-lox system to express *Fmr1* only in excitatory or inhibitory neurons [130, 131] will enable researchers to separate the roles of FMRP in regulating development of excitatory versus inhibitory circuits.

## 6. Conclusions

Neuronal plasticity establishes and maintains connectivity and defines the operational range and coding capacity of neuronal networks. In FXS, the absence of a single protein, FMRP, perturbs the balance in a diverse array of plasticity mechanisms from Hebbian to homeostatic, which alters the establishment and maintenance of this operational range and coding capacity of FXS networks in a developmentally and regionally specific way. Most of these mechanisms likely involve dysregulation of processes downstream of GpI mGluR signaling as a result of the absence of a key transcriptional regulator in FMRP. However, as researchers begin to investigate early developmental processes in FXS, a more diverse role of FMRP in plasticity mechanisms begins to emerge that may provide new avenues for treatment that alter pathological plasticity underlying the disease progression. As Krueger and Bear describe in a recent review (2011), the key to treating a developmental disorder like FXS relies on treatment at the critical developmental time point where developmental progression diverges from the norm [125]. Unfortunately circuit alterations precede behavioral signs of pathological disturbances. By investigating dysfunctional plasticity related to the loss of FMRP earlier in development, we can better identify that point of divergence and design treatments that not only correct abnormal plasticity and thereby their behavioral correlates but also minimize the establishment of the plasticity-deficient networks in the first place.
